# Supplementary material for: Altered function and differentiation of age-associated B cells contribute to the female bias in lupus mice
Source: Nat Commun. 2021 Aug 10;12:4813. doi: 10.1038/s41467-021-25102-8 (PMC8355159; doi:10.1038/s41467-021-25102-8)
Supplement: Supplementary file 1 — Supplementary Information [file 41467_2021_25102_MOESM1_ESM.pdf]

Supplementary Information.

Differences in age-associated B cells promote the female sex bias in lupus pathogenesis

Edd Ricker[1,2], Michela Manni[1], Danny Flores-Castro[1], Daniel Jenkins[1], Sanjay Gupta[1], Juan Rivera-Correa[1,2], Wenzhao Meng[3], Aaron M. Rosenfeld[3], Tania Pannellini[4], Mahesh Bachu[5], Yurii Chinenov[6], Peter K. Sculco[7], Rolf Jessberger[8], Eline T. Luning Prak[3], Alessandra B. Pernis[1,2,6, 9 *]

[1]Autoimmunity and Inflammation Program, Hospital for Special Surgery, New York, NY, USA

[2]Department of Microbiology and Immunology, Weill Cornell Medicine, New York, NY, USA

[3]Department of Pathology and Laboratory Medicine, Perelman School of Medicine, Philadelphia, PA, USA

[4]Research Division and Precision Medicine Laboratory, Hospital for Special Surgery, New York, NY, USA

[5]Arthritis and Tissue Degeneration Program, Hospital for Special Surgery, New York, NY, USA

[6]David Z. Rosensweig Genomics Research Center, Hospital for Special Surgery, New York, NY, USA

[7]Department of Orthopedic Surgery, Hospital for Special Surgery, New York, NY, USA

[8]Institute of Physiological Chemistry, Technische Universitat, Dresden, Germany.

[9]Department of Medicine, Weill Cornell Medicine, New York, NY, USA

*E-mail: pernisa@hss.edu

Supplementary Figure 1, related to Figure 1.

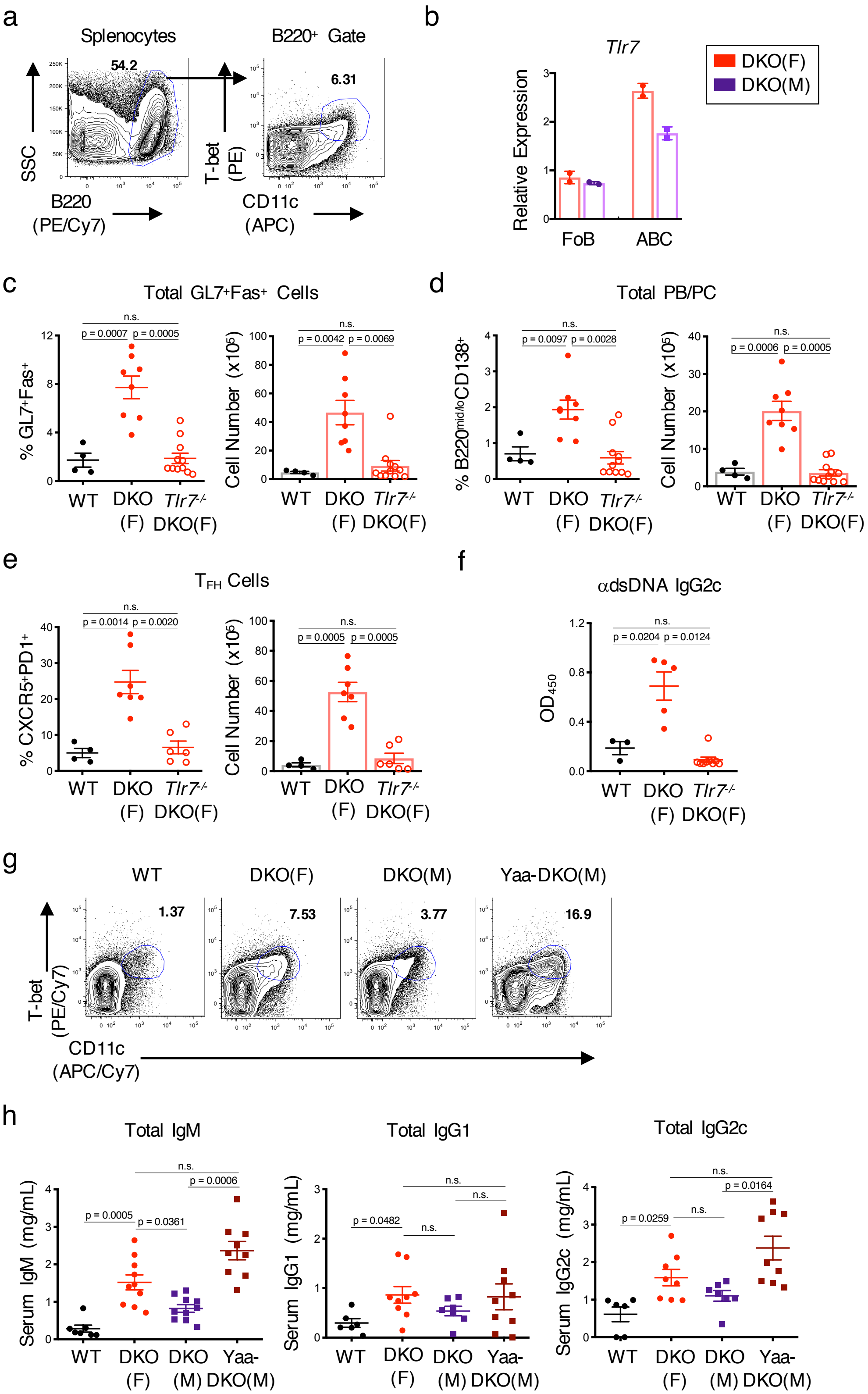

Supplementary Figure 1, related to Figure 1. (A) Gating schematic showing the identification of $CD11c^{+}Tbet^{+}$ ABCs (gated on $B220^{+}$ splenocytes) as in Fig1A. (B) Representative RT-qPCR of *Tlr7* expression in sorted ABCs ($B220^{+}CD19^{+}CD11c^{+}CD11b^{+}$) or follicular B cells (FoB; $B220^{+}CD19^{+}CD11c^{-}CD11b^{-}CD23^{+}$) from aged (24+wk) female DKO (F) or male DKO (M) mice. Data representative of 2 independent experiments and show mean +/- SD; n=2 technical replicates per experiment. (C-E) Quantifications of total $GL7^{+}Fas^{+}$ B cells (*C;* $B220^{+}$ $GL7^{+}Fas^{+}$), total PB/PC (*D;* $B220^{mid/lo}CD138^{+}$), and T_{FH} cells (*E;* $CD4^{+}$ $PD1^{hi}CXCR5^{+}$) from the spleens of aged (24+wk) female C57BL/6 (WT), DKO(F), and female *Tlr7*-deficient (*$Tlr7^{-/-}$*.DKO(F)) mice. Data show mean +/- SEM; for C-D, n=4 for WT, n=8 for DKO(F), n=11 for *$Tlr7^{-/-}$*.DKO(F); for E, n=4 for WT, n=7 for DKO(F), n=6 for *$Tlr7^{-/-}$*.DKO(F) over independent 6 experiments; p-values by Brown-Forsythe and Welch ANOVA followed by Games-Howell's test for multiple comparisons. (F) Pooled ELISA data for anti-dsDNA IgG2c autoantibodies in serum from the indicated mice as in (*C-E*). Data show mean +/- SEM; n=3 for WT, n=5 for DKO(F), n=9 for *$Tlr7^{-/-}$*.DKO(F); p-values by Brown-Forsythe and Welch ANOVA followed by Games-Howell's test for multiple comparisons. (G) Representative FACS plots of $CD11c^{+}Tbet^{+}$ ABCs from the spleens of aged (24+wk) WT, DKO(F), DKO(M), and Yaa-DKO(M) mice as in Fig. 1D. (H) ELISA data for IgM, IgG1, and IgG2c antibodies in serum from the indicated aged (24+wk) mice. Data show mean +/- SEM; for IgM, n=7 for WT, n=10 for DKO(F), n=10 for DKO(M), n=9 for Yaa-DKO(M); for IgG1, n=6 for WT, n=9 for DKO(F), n=7 for DKO(M), n=9 for Yaa-DKO(M); for IgG2c, n=6 for WT, n=8 for DKO(F), n=7 for DKO(M), n=9 for Yaa-DKO(M) over 7 independent experiments; p-value by Brown-Forsyth and Welch ANOVA followed by Games-Howell's test for multiple comparisons.

Supplementary Figure 2, related to Figure 2.

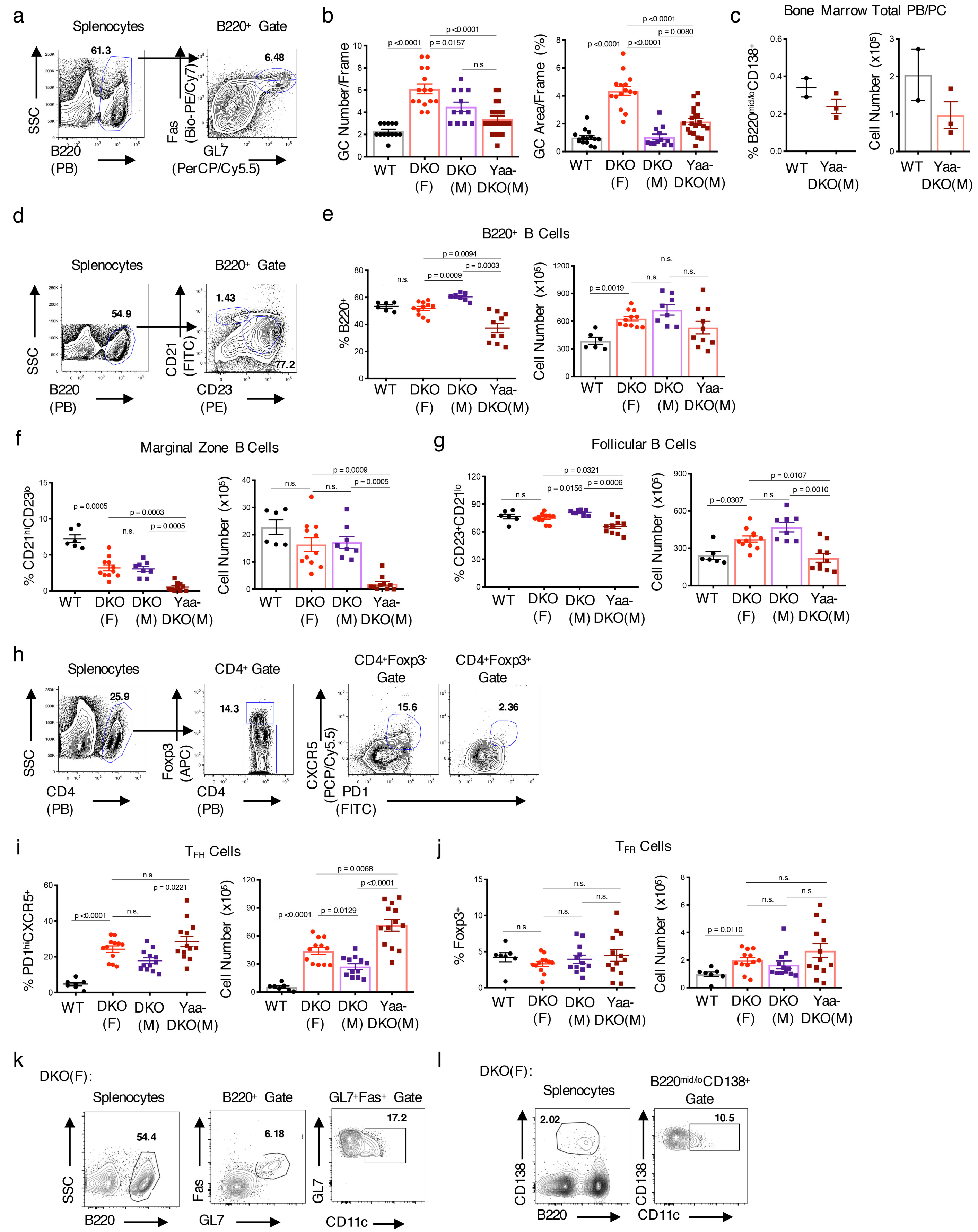

Supplementary Figure 2, related to Figure 2. (A) Gating schematic showing the identification of $GL7^{+}Fas^{+}$ GC B cells (gated on $B220^{+}$ splenocytes) from a DKO(F) mouse as in Fig2A. (B) Quantification of immunofluorescence images in Fig. 2B. Plots show GC count per frame *(left)* and GC area per frame (% $GL7^{+}B220^{+}$) (*right*). Data pooled from at least 4 frames for at least 2 mice per genotype and show mean +/- SEM; n=15 for WT and DKO(F), n=12 for DKO(M), n=19 for Yaa-DKO(M) over 3 independent experiments; p-value by Brown-Forsythe and Welch ANOVA followed by Games-Howell's test for multiple comparisons. (C) Quantifications of FACS data showing total bone marrow PB/PCs ($B220^{mid/lo}CD138^{+}$). Data show mean +/- SEM; n=2 for WT, n=3 for Yaa-DKO(M). (D) Gating schematic showing the identification of marginal zone B cells ($B220^{+}CD21^{hi}CD23^{lo}$ splenocytes) and follicular B cells ($B220^{+}CD23^{+}CD21^{lo}$ splenocytes) from a DKO(F) mouse. (E-G) Quantifications of total B cells (*E*), marginal zone B cells (*F*), and follicular B cells (*G*) from the indicated mice. Data show mean +/- SEM; n=6 for WT, n=11 for DKO(F), n=8 for DKO(M), n=10 for Yaa-DKO(M) over 6 independent experiments; p-value by Brown-Forsythe and Welch ANOVA followed by Games-Howell's test for multiple comparisons. (H) Gating schematic showing the identification of T_{FH} ($CD4^{+}Foxp3^{-}$ $PD1^{hi}CXCR5^{+}$ splenocytes) and T_{FR} ($CD4^{+}Foxp3^{+}$ $PD1^{hi}CXCR5^{+}$ splenocytes) cells. (I-J) Quantifications of T_{FH} (*I*) and T_{FR} (*J*) cells from the indicated mice. Data show mean +/- SEM; for (*I*), n=7 for WT, n=12 for DKO(F) and DKO(M), n=13 for Yaa-DKO(M); p-value by Brown-Forsythe and Welch ANOVA followed by Games-Howell's test for multiple comparisons. (K-L) Representative FACS plots showing the gating schematic for $CD11c^{+}$ $GL7^{+}Fas^{+}$ cells in Fig. 2F (*K*) and for $CD11c^{+}$ PB/PC in Fig. 2G (*L*) from DKO(F) mice.

Supplementary Figure 3, related to Figure 3.

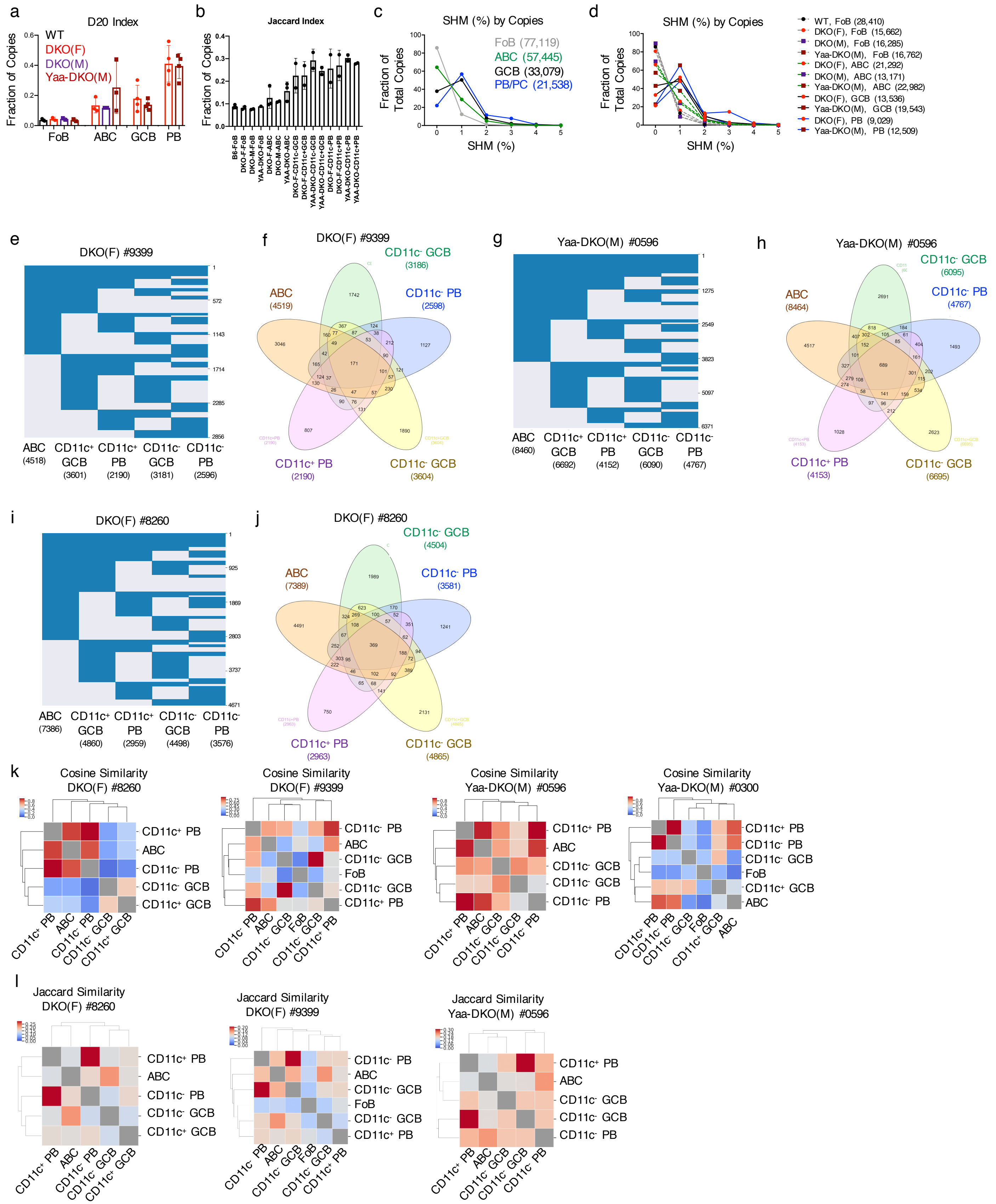

Supplementary Figure 3, related to Figure 3. (A) D20 index of each mouse strain/subset combination. Data show mean +/- SD; n=2-4 mice. (B) Plot showing the level of overlap between clones in different strains/subsets. For each comparison, each clone was only counted once (no weighting for clone size) and functional overlap was computed using the Jaccard Index. Data show mean +/- SD; n=2-3 mice. (C-D) SHM data with sequence data weighted by copies (clone size). (E,G,I) Plot showing clones (rows) that overlap between at least two B cell subsets (columns). Numbers along the right side of the plot indicate clone counts. Data from the analysis of subsets from a single DKO female or Yaa-DKO male mouse as indicated. (F,H,J) Venn diagram showing clonal overlap where numbers indicate clone counts in the different subset interactions. Data from the analysis of subsets from a single DKO female or Yaa-DKO male mouse as indicated. (K) Heatmap showing Cosine similarity. Data from the analysis of a single DKO female or Yaa-DKO male mouse as indicated. (L) Heatmap showing Jaccard similarity (fraction of clones that overlap between different two-subset comparisons). Diagonal values are excluded for scaling. Data from the analysis of a single DKO female or Yaa-DKO male mouse as indicated.

Supplementary Figure 4, related to Figure 4.

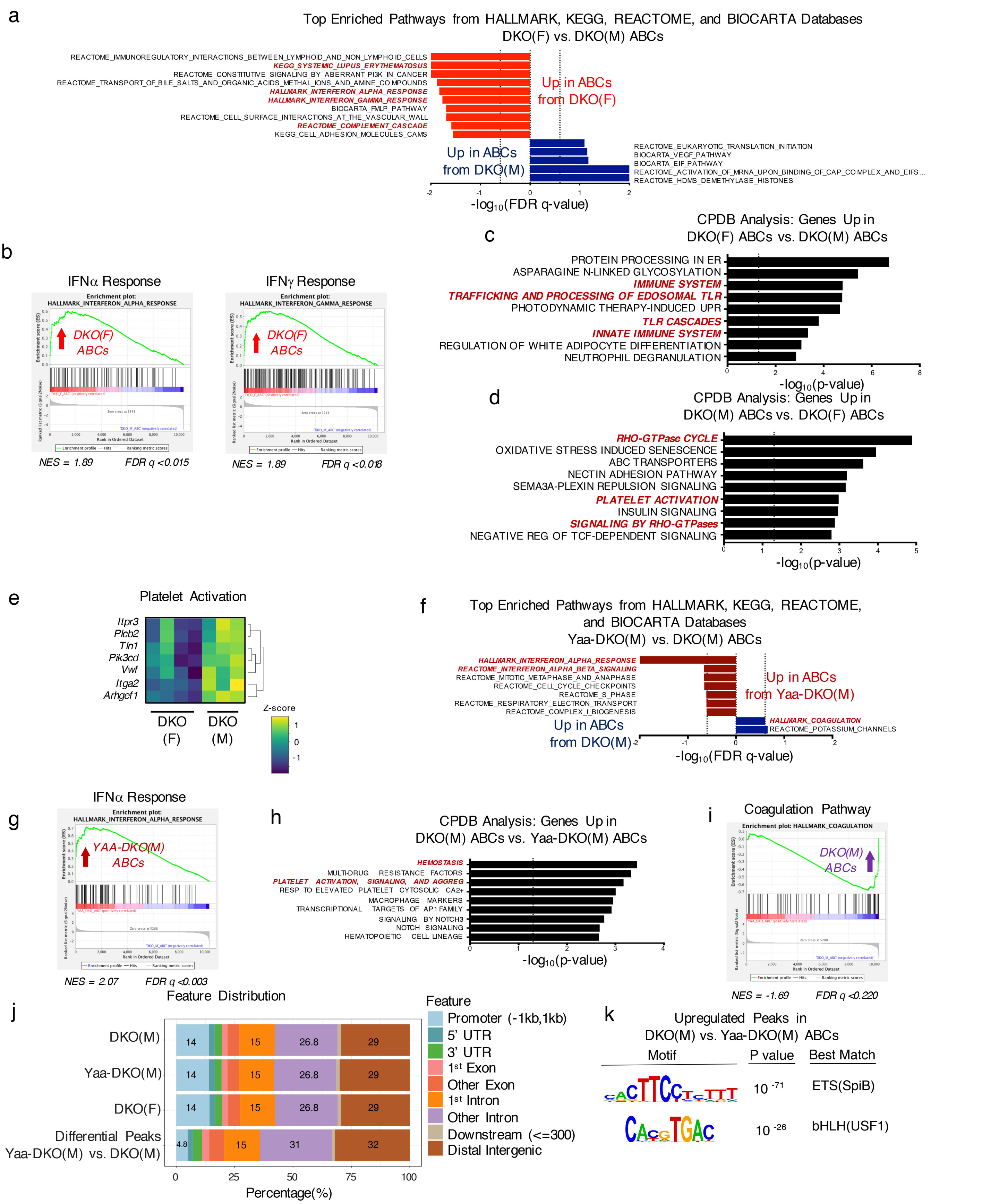

Supplementary Figure 4, related to Figure 4.

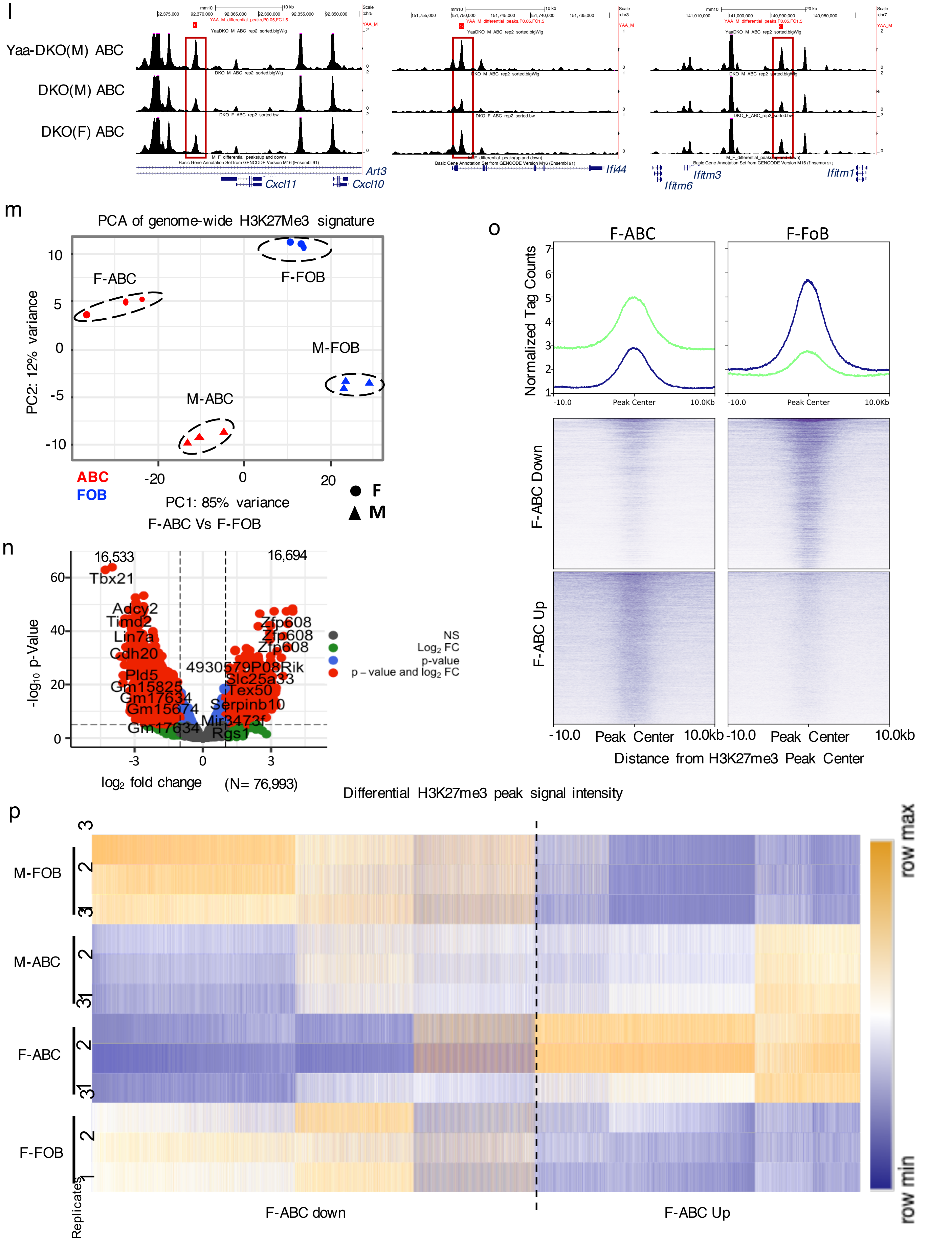

Supplementary Figure 4, related to Figure 4. (A-I) RNA-seq was performed on sorted ABCs (CD19$^+$B220$^+$CD11c$^+$CD11b$^+$) from DKO(F), DKO(M), and Yaa-DKO(M) mice as in Fig. 4. (A) Plot showing the top pathways enriched in ABCs from DKO(F) *(red)* and DKO(M) *(blue)* mice as determined by GSEA using the HALLMARK, KEGG, REACTOME, and BIOCARTA databases. Dotted lines show significance thresholds at FDR q <0.25. (B) Plot showing enrichment of the HALLMARK_INTERFERON_ALPHA_RESPONSE and HALLMARK_ INTERFERON_GAMMA_ RESPONSE pathways in ABCs from DKO(F) mice. (C-D) Plots showing the top over-represented pathways comprising the genes significantly upregulated p <0.01 after Benhamini-Hochberg false discovery rate (FDR) was used to correct for multiple comparisons) in ABCs from DKO(F) mice as compared to DKO(M) mice *(C)* or in ABCs from DKO(M) mice as compared to DKO(F) mice *(D)* using Consensus Pathway Database (CPDB). (E) Heatmap showing differentially expressed genes in the KEGG_PLATELET_ACTIVATION pathway. (F) Plot showing the top pathways enriched in ABCs from Yaa-DKO(M) *(maroon)* and DKO(M) *(blue)* mice as determined by GSEA using the HALLMARK, KEGG, REACTOME, and BIOCARTA databases. Dotted lines show significance thresholds at FDR q <0.25. (G) Table showing the top over-represented pathways comprising the genes significantly upregulated in ABCs from DKO(M) mice as compared to Yaa-DKO(M) mice using CPDB. (H) Plot showing the enrichment (FDR q <0.25) of the HALLMARK_INTERFERON_ALPHA_RESPONSE geneset in ABCs from Yaa-DKO(M) mice. (I) Plot showing the enrichment of the HALLMARK_COAGULATION geneset in DKO(M) ABCs as compared to Yaa-DKO(M) ABCs. (J) Plot showing the genomic distribution of peaks from ATACseq. (K) Motif enrichment analysis in ATAC-seq peaks that are significantly upregulated (logFC >1.5; p <0.05 after Benhamini-Hochberg false discovery rate (FDR) was used to correct for multiple comparisons) in ABCs from DKO(M) mice. (L) Representative tracks showing accessible chromatin at the *Cxcl* cluster from DKO(F), DKO(M), and Yaa-DKO ABCs. (M) Principal component analysis of global H3K27me3 peaks. PC1 (85% variance) demarcates samples based on B cell phenotype and PC2 (12% variance) based on sex from CUT&RUN analysis. (N) The differential analysis are carried out according to the DESeq2 model and package. For this analysis, a Benjamini-Hochberg p-value adjustment was performed, and the level of controlled false positive rate was set to 0.05. The raw p-value were obtained from the Wald-statistical test. Volcano plot showing differential H3K27me3 peaks between ABCs and FoBs from DKO(F) mice. A total of 76,993 peaks were identified of which 16,533/16,694 peaks were down/up-regulated by 2-fold and p <0.05). (O) Average profile plot of H3K27me3 signal extended to -/+10 kb from the peak-center of 16,533 peaks in either downregulated in ABCs (*blue line*) or of 16,694 peaks upregulated in ABCs over FoBs from DKO(F) mice. Y-axis represents average tag density normalized to reads per million. A heatmap of the differential peaks of ABCs vs FoBs showing the spread of H3K27me3 signal from the peak center in ABC and FoB samples. (P) Heatmap depiction of tag densities of H3K27me3 peaks for down and upregulated peaks for individual replicates for ABCs and FoBs from male and female DKOs.

Supplementary Figure 5, related to Figure 5.

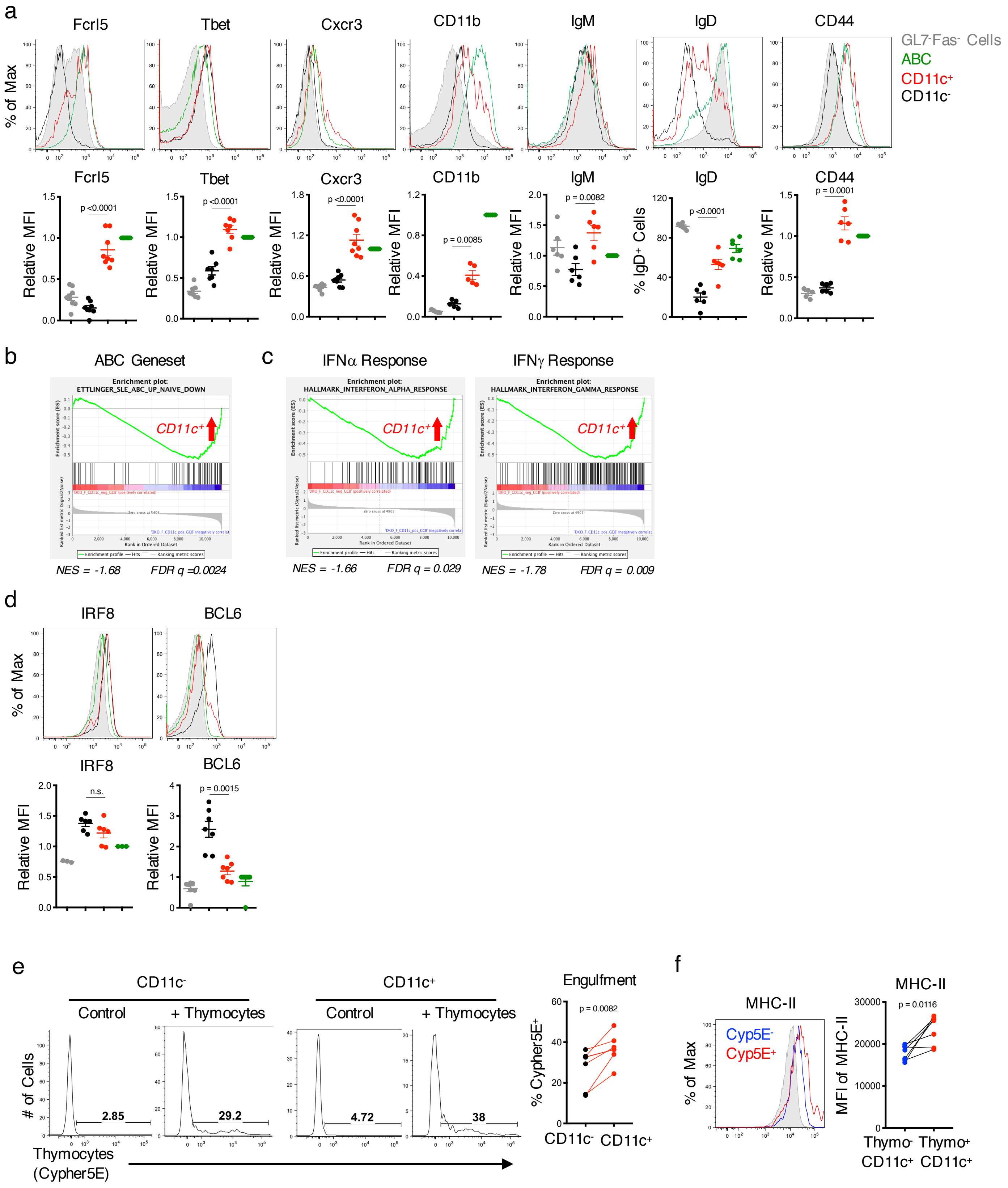

Supplementary Figure 5, related to Figure 5. (A) Representative histograms and quantifications of Fcrl5, Tbet, Cxcr3, CD11b, IgM, IgD, and CD44 expression in CD11c$^+$ CD19$^+$GL7$^+$Fas$^+$ cells (CD11c$^+$) (*red*), CD11c$^-$ CD19$^+$GL7$^+$Fas$^+$ cells (CD11c$^-$) *(black)*, and CD19$^+$CD11c$^+$CD11b$^+$ (ABCs) *(green)* from aged (24+wk) female DKO (F) mice. CD19$^+$Fas$^-$ GL7$^-$ cells are shown as a control *(gray)*. Data show mean +/- SEM; n=5 for CD11b, n=6 for IgM, IgD and CD44, n=7 for Tbet, n=8 for FcRL5 and CXCR3 over 3-5 independent experiments; p-value by paired two-tailed t-tests. (B) Plot showing the enrichment of an ABC geneset from SLE patients in CD11c$^+$ B220$^+$GL7$^+$CD38^{lo} cells as in Fig. 5A[15]. (C) Plot showing the enrichment of the HALLMARK_INTERFERON_ALPHA_RESPONSE and the HALLMARK_ INTERFERON_GAMMA_RESPONSE genesets in CD11c$^+$ B220$^+$GL7$^+$CD38^{lo} cells as in Fig. 5. (D) Representative histograms and quantifications of IRF8 and BCL6 in the indicated populations from aged (24+wk) DKO(F) mice. Data show mean +/- SEM; n=6 for IRF8, n=7 for BCL6 over 4 independent experiments; p-value by paired two-tailed t-tests. (E-F) Splenocytes from aged (24+wk) male and female DKO mice were co-cultured for 3hr with Cypher5E-labeled thymocytes following induction of apoptosis with 50mM Dexamethasone. (E) Representative histograms and quantifications showing the percentage of CD11c$^+$ and CD11c$^-$ CD19$^+$GL7$^+$Fas$^+$ cells that engulfed apoptotic thymocytes (Cypher5E$^+$); Data pooled from 4 DKO(F), 1 DKO(M), and 1 YAA-DKO(M) mice; p-value by paired two-tailed t-test. (F) Representative histogram and quantification of MHC-II expression in CD11c$^+$ CD19$^+$GL7$^+$Fas$^+$ B cells that engulfed *(red*) or did not engulf *(blue)* apoptotic thymocytes. Data representative of and/or pooled from 6 mice as in Fig. S5E; p-value by paired two-tailed t-test.

Supplementary Figure 6, related to Figure 6.

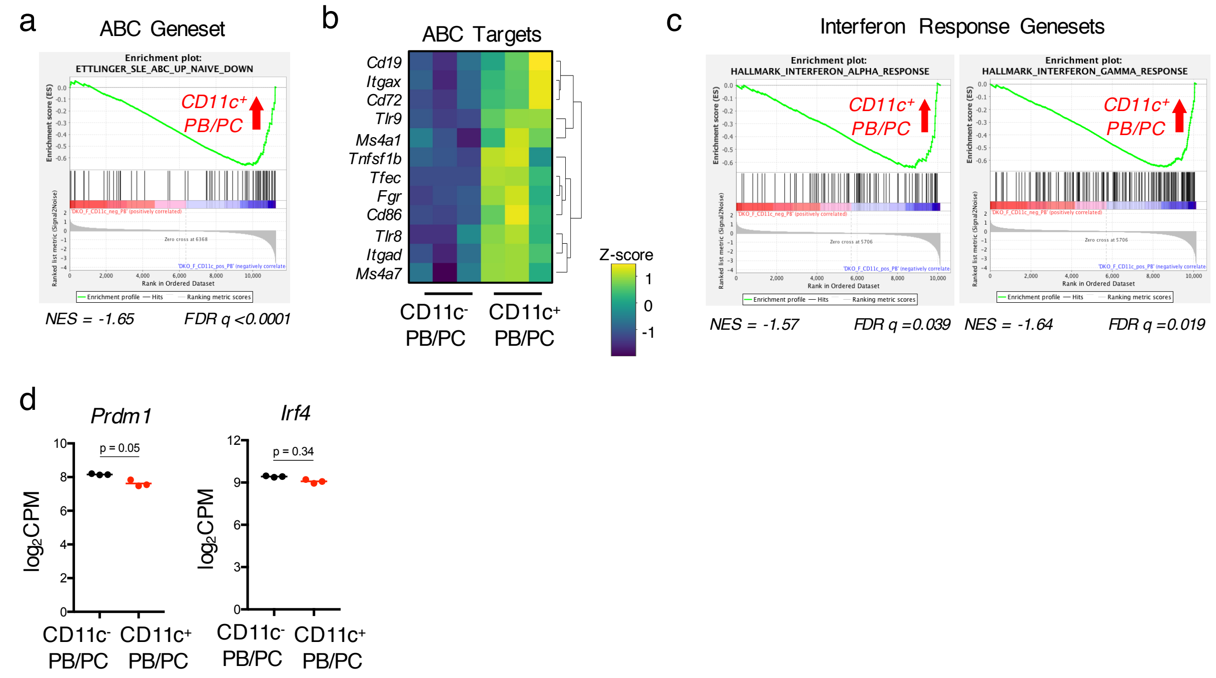

Supplementary Figure 6, related to Figure 6. RNA-seq was performed on sorted CD11c^{+} and CD11c^{-} PB/PCs (CD138^{+}TACI^{+}) from aged (24+wk) female DKO (F) mice as in Fig. 6A. (A) Plot showing the enrichment of an ABC geneset from SLE patients in CD11c^{+} PB/PCs[15]. (B) Heatmap showing the differential expression of ABC target genes in CD11c^{+} and CD11c^{-} PB/PCs. (C) Plot showing the enrichment of the HALLMARK_INTERFERON_ALPHA_ RESPONSE and the HALLMARK_ INTERFERON_GAMMA_ RESPONSE genesets in CD11c^{+} PB/PCs. (D) Plots showing the normalized log_2CPM counts for *Prdm1* and *Irf4* in CD11c^{+} and CD11c^{-} PB/PCs from Fig. 6A.

Supplementary Figure 7, related to Figure 8.

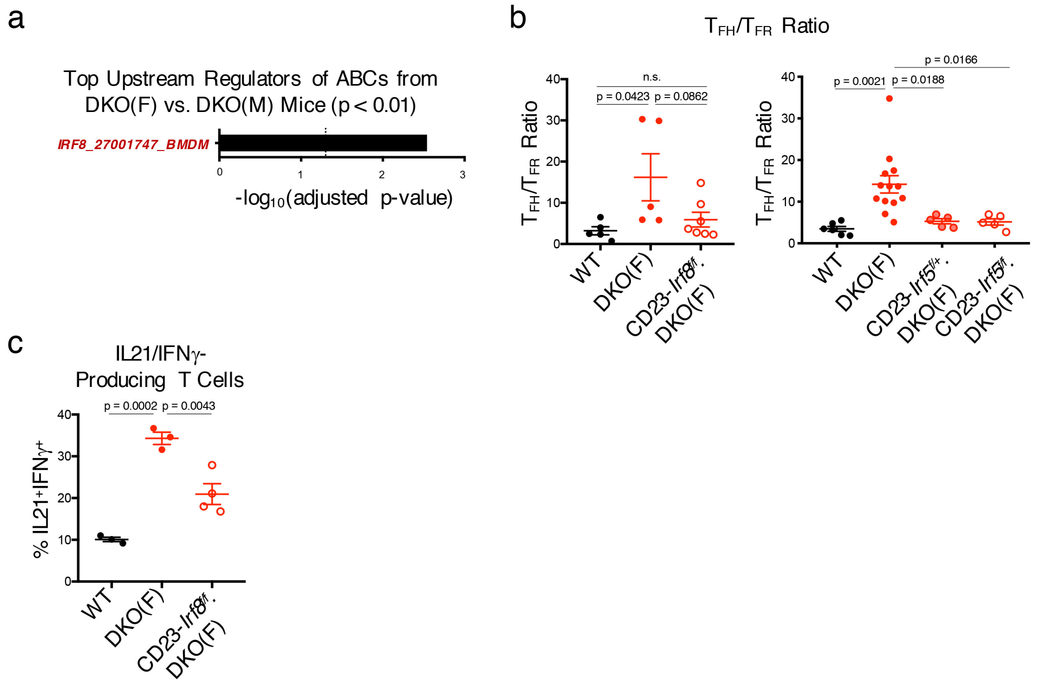

Supplementary Figure 7, related to Figure 8. (A) Plot showing Enrichr analysis of potential upstream regulators for the geneset upregulated in ABCs from aged (24wk+) female DKO(F) as compared to male DKO(M) mice. (B) Quantifications showing the ratio of T_{FH} to T_{FR} cells from aged (24wk+) female C57BL/6 (WT), female DKO(F), female CD23-Cre.*Irf8*$^{f/f}$.DKO(F), female CD23-Cre.*Irf5*$^{f/+}$.DKO(F), and female CD23-Cre.*Irf5*$^{f/f}$.DKO(F) mice. Data show mean +/- SEM; n=5/6 for WT, n=5/13 for DKO(F), n=7 for CD23-*Irf8*.DKO(F), n=5 for CD23-*Irf5*$^{f/+}$.DKO(F) and CD23-*Irf5*$^{f/f}$.DKO(F) over 6-8 independent experiments; p-value by 1-way ANOVA followed by Tukey's test for multiple comparisons. (C) Quantifications showing the production of IL21 and IFNg in T cells from the indicated aged (24wk+) mice. Data show mean +/- SEM; n=3 for WT and DKO(F), n=4 for CD23-*Irf8*.DKO(F) over 3 independent experiments; p-value by 1-way ANOVA followed by Tukey's test for multiple comparisons.

Supplementary Figure 8, related to Figure 9.

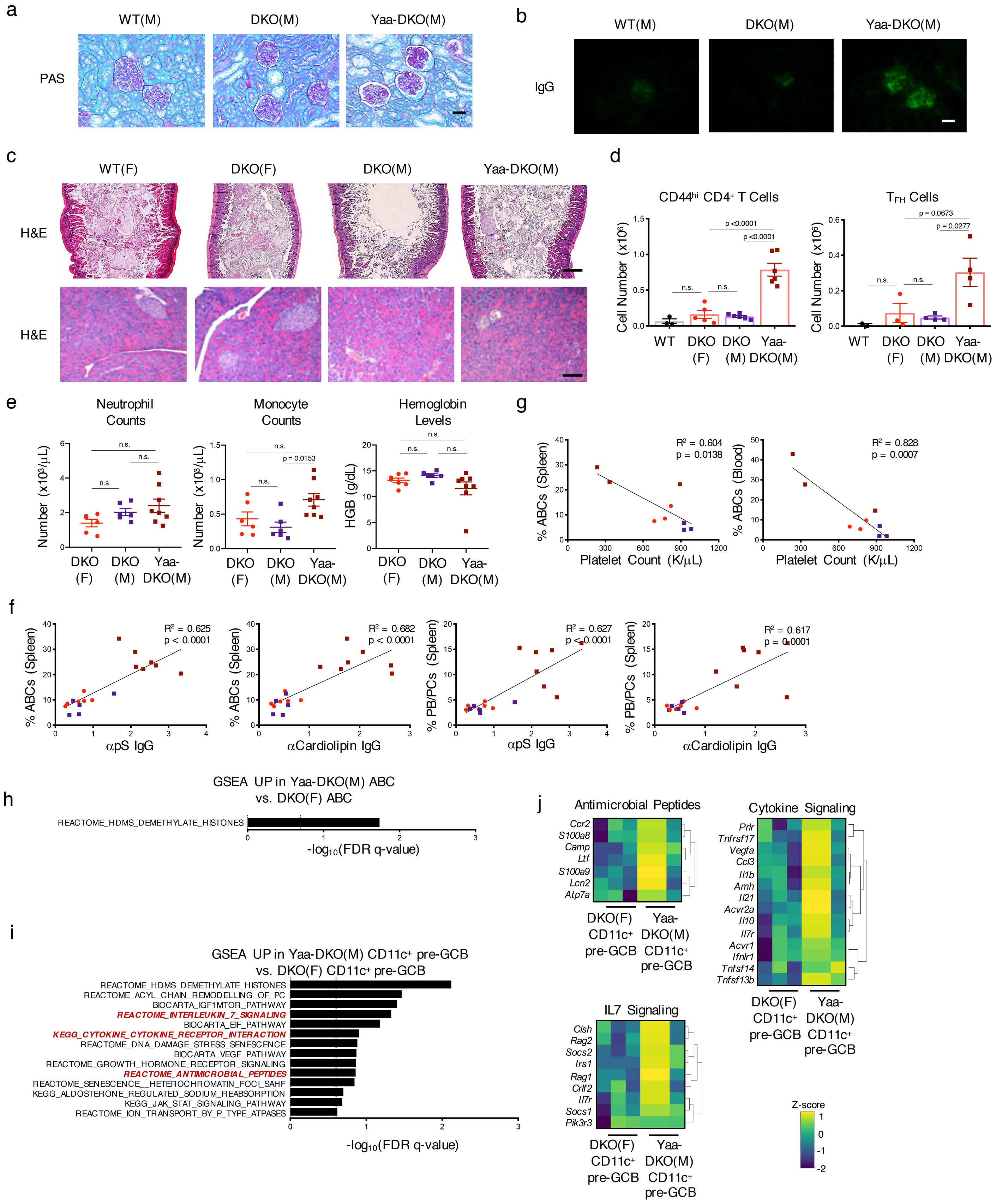

Supplementary Figure 8, related to Figure 9. (A) Representative images of PAS staining in kidneys from the aged (24wk+) female C57BL/6 (WT), male DKO (M), and male Yaa-DKO (M) mice. Data representative of 4 kidneys per genotype. Bars show 50 μm. (B) Representative images of IgG deposition in kidneys from the indicated mice. Data representative of 3 kidneys per genotype. (C) Representative H&E images of colon *(top)* and pancreas *(bottom)* from the indicated mice. Data representative of 2 WT, 4 female DKO (F), 4 DKO(M), and 4 Yaa-DKO(M) mice. Bars show 50 μm. (D) Quantifications showing the numbers of $CD44^{hi}$ $CD4^{+}$ T cells and T_{FH} cells (CD4+ $PD1^{hi}CXCR5^{+}$) in the lungs of the indicated mice. Data show mean +/- SEM; n=2-3 for WT, n=3-5 for DKO(F), n=4-6 for DKO(M), and n=4-6 for Yaa-DKO(M) over 2 independent experiments; p-value by 1-way ANOVA followed by Tukey's test for multiple comparisons. (E) Plots showing the numbers of neutrophils and monocytes and the levels of hemoglobin in the blood from the indicated mice. Data show mean +/- SEM; n=6 for DKO(F) and DKO(M), n=8 for Yaa-DKO(M); p-value by 1-way ANOVA followed by Tukey's test for multiple comparisons. (F) Plots showing the correlations between the frequencies of ABCs or PB/PCs in the spleen and serum levels of anti-pS IgG and anti-Cardiolipin IgG antibodies. Data from DKO(F) (*red circles*), DKO(M) (*purple squares*), and Yaa-DKO(M) *(maroon squares*) are shown; n=6 for DKO(F), n=5 for DKO(M), n=7 for Yaa-DKO(M); p-value by Pearson correlation. (G) Plots showing the correlations between platelet counts and the frequencies of $CD11c^{+}CD11b^{+}$ ABCs in the spleen or blood. (H) Plots showing the top pathways enriched in ABCs from Yaa-DKO(M) mice as compared to those from DKO(F) mice as determined by GSEA. Dotted line indicates significance threshold at FDR q <0.25. (I-J) RNA-seq was performed on sorted $CD11c^{+}$ $GL7^{+}CD38^{lo}$ (pre-GC) B cells from Yaa-DKO(M) mice. (I) Plots showing the top pathways enriched in $CD11c^{+}$ pre-GC B cells from Yaa-DKO(M) mice as compared to those from DKO(F) mice as determined by GSEA. Dotted line indicates significance threshold at FDR q <0.25. (J) Heatmaps showing the expression of genes enriching the antimicrobial peptide, cytokine signaling, and IL7 signaling genesets in Yaa-DKO(M) $CD11c^{+}$ pre-GC B cells.

Supplementary Figure 9

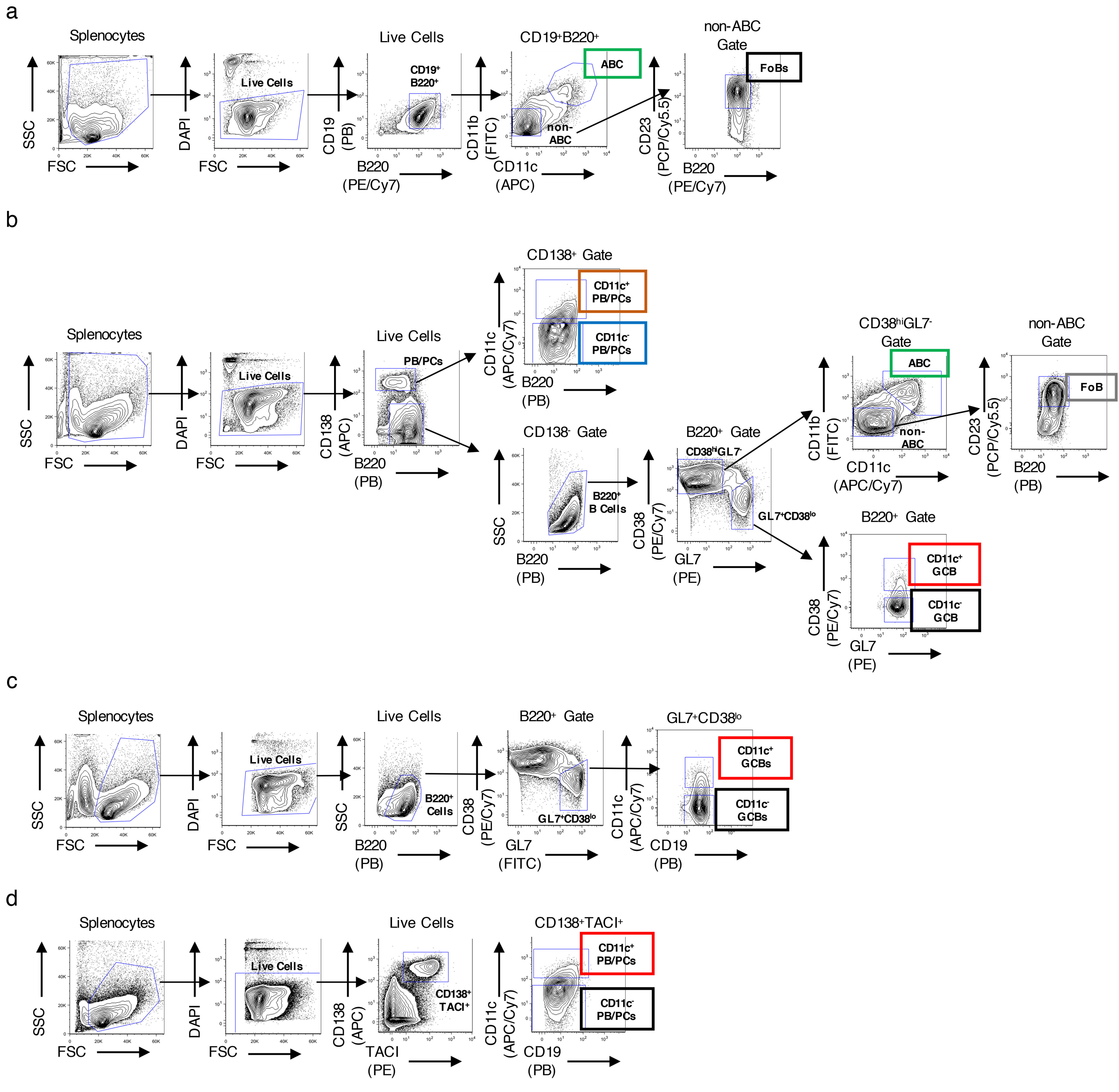

Supplementary Figure 9. (A) Gating strategy for ABC (DAPI$^-$CD19$^+$B220$^+$CD11b$^+$CD11c$^+$) and FoB (DAPI$^-$CD19$^+$B220$^+$CD11b$^-$CD11c$^-$CD23^{hi}) populations depicted in Fig. 1B,E-F, Fig. 4 and Fig. S8H. (B) Gating strategy for ABC (DAPI$^-$CD138$^-$B220$^+$ CD38^{hi}GL7$^-$CD11b$^+$CD11c$^+$), FoB (DAPI$^-$CD138$^-$B220$^+$CD38^{hi}GL7$^-$CD11b$^+$CD11c$^+$CD23^{hi}), CD11c+ PB/PC (DAPI$^-$CD138$^+$B220$^{mid/lo}$CD11c$^+$), CD11c- PB/PC (DAPI$^-$CD138$^+$B220$^{mid/lo}$ CD11c$^-$), CD11c+ GCB (DAPI$^-$CD138$^-$B220$^+$CD38^{lo}GL7$^+$CD11c$^+$), and CD11c- GCB (DAPI$^-$CD138$^-$B220$^+$ CD38^{lo}GL7$^+$CD11c$^-$) populations depicted in Fig. 3. (C) Gating strategy for CD11c$^+$ GCB (DAPI$^-$ B220$^+$CD38^{lo}GL7$^+$CD11c$^+$) and CD11c$^-$ GCB (DAPI$^-$ B220$^+$CD38^{lo}GL7$^+$CD11c$^-$) populations as depicted in Fig. 5 and Fig. S8I-J. (D) Gating strategy for CD11c$^+$ PB/PC (DAPI$^-$CD138$^+$TACI$^+$CD11c$^+$) and CD11c$^-$ PB/PC (DAPI$^-$CD138$^+$TACI$^+$CD11c$^+$) as depicted in Fig. 6 and Fig. 9J-K.

Supplementary Table 1.

Table S1. ATACseq Metrics

SampleID	ReadsInPeaks	ReadsOutPeaks	ReadsTotal	FRIP
F_DKO_ABC_Rep1	81242235	62367319	143609554	0.56571609
F_DKO_ABC_Rep2	76258833	84410832	160669665	0.47463118
M_DKO_ABC_Rep1	79714424	94817706	174532130	0.45673209
M_DKO_ABC_Rep2	78269373	88215789	166485162	0.47012822
YAA_DKO_ABC_Rep1	78804122	115187436	193991558	0.40622449
YAA_DKO_ABC_Rep2	89185104	65879978	155065082	0.57514627

Supplementary Table 1. ATACseq Metrics. Table showing the read totals and distributions from the ATAC-seq analysis in Fig. 4.

Supplementary Table 2.

Table S2. qPCR Primers

Primer	Forward	Reverse
Mouse Ciita	Qiagen (PPM03368F-200)	Qiagen (PPM03368F-200)
Mouse Tlr7	Qiagen (PPM04208A-200)	Qiagen (PPM04208A-200)
Mouse Ppia	5'-TTG CCA TTC CTG GAC CCA AA-3'	5'-ATG GCA CTG GCG GCA GGT CC-3'
Mouse Il1b	5' -AGC TTC CTT GTG CAA GTG TCT-3'	5' -GAC AGC CCA GGT CAA AGG TT-3'

Table S2. ChIP-qPCR Primers

Primer	Forward	Reverse
Mouse Cxcl cluster	5'-AGA ACC GAC GCC ATC TGA AT-3'	5'-GGC TCC TTG ACT AGC CTA ACC-3'

Supplementary Table 2. Primer Information. Table showing the primers used for RT-qPCR and ChIP-qPCR.
