# Supplementary material for: Altered function and differentiation of age-associated B cells contribute to the female bias in lupus mice
Source: Nat Commun. 2021 Aug 10;12:4813. doi: 10.1038/s41467-021-25102-8 (PMC8355159; doi:10.1038/s41467-021-25102-8)
Supplement: Supplementary file 3 — Description of Additional Supplementary Files [file 41467_2021_25102_MOESM3_ESM.pdf]

Description of Additional Supplementary Files

File Name: Supplementary Data 1
Description: Differentially Expressed Peaks between ABCs in DKO(F) vs DKO(M).

File Name: Supplementary Data 2
Description: Differentially Expressed Peaks between ABCs in Yaa-DKO vs. DKO(M).

File Name: Supplementary Data 3
Description: CUT&RUN Dataset from DKO(M) and DKO(F) FoBs and ABCs.

File Name: Supplementary Data 4
Description: Metadata Table for BCR-seq.
