# Supplementary material for: Altered function and differentiation of age-associated B cells contribute to the female bias in lupus mice
Source: Nat Commun. 2021 Aug 10;12:4813. doi: 10.1038/s41467-021-25102-8 (PMC8355159; doi:10.1038/s41467-021-25102-8)
Supplement: Supplementary file 8 — Reporting Summary [file 41467_2021_25102_MOESM8_ESM.pdf]

nature research

Corresponding author(s): Pernis, AB

Last updated by author(s): May 16, 2021

Reporting Summary

Nature Research wishes to improve the reproducibility of the work that we publish. This form provides structure for consistency and transparency in reporting. For further information on Nature Research policies, see our Editorial Policies and the Editorial Policy Checklist.

Statistics

For all statistical analyses, confirm that the following items are present in the figure legend, table legend, main text, or Methods section.

n/a	Confirmed	
☐	☒	The exact sample size (*n*) for each experimental group/condition, given as a discrete number and unit of measurement
☐	☒	A statement on whether measurements were taken from distinct samples or whether the same sample was measured repeatedly
☐	☒	The statistical test(s) used AND whether they are one- or two-sided<br>*Only common tests should be described solely by name; describe more complex techniques in the Methods section.*
☐	☒	A description of all covariates tested
☐	☒	A description of any assumptions or corrections, such as tests of normality and adjustment for multiple comparisons
☐	☒	A full description of the statistical parameters including central tendency (e.g. means) or other basic estimates (e.g. regression coefficient) AND variation (e.g. standard deviation) or associated estimates of uncertainty (e.g. confidence intervals)
☐	☒	For null hypothesis testing, the test statistic (e.g. *F*, *t*, *r*) with confidence intervals, effect sizes, degrees of freedom and *P* value noted<br>*Give P values as exact values whenever suitable.*
☒	☐	For Bayesian analysis, information on the choice of priors and Markov chain Monte Carlo settings
☒	☐	For hierarchical and complex designs, identification of the appropriate level for tests and full reporting of outcomes
☒	☐	Estimates of effect sizes (e.g. Cohen's *d*, Pearson's *r*), indicating how they were calculated

Our web collection on statistics for biologists contains articles on many of the points above.

Software and code

Policy information about availability of computer code

Data collection

Provide a description of all commercial, open source and custom code used to collect the data in this study, specifying the version used OR state that no software was used.

Data analysis

For RNAseq analysis, read quality was assessed and adaptors trimmed using FASTP(71). Reads were then mapped to the mouse genome (mm10) and reads in exons were counted against Gencode v27 with STAR2.6 Aligner(72). Differential gene expression analysis was performed in R using edgeR3.24.3. Genes with low expression levels (<2 counts per million in at least one group) were filtered from all downstream analyses. Differential expression was estimated using quasi-likelihood framework. Benhamini-Hochberg false discovery rate (FDR) procedure was used to correct for multiple testing. Genes with an unadjusted p-value less than 0.01 were considered differentially expressed. Downstream analyses were performed in R using a visualization platform built with Shiny developed by bioinformaticians at the David Z. Rosensweig Genomics Research Center at HSS.

For ATAC-seq analyses, paired-end 50bp sequences were generated from samples with an Illumina HiSeq2500 and, following adapter trimming with FastP, were aligned against mouse genome (mm10) using bowtie2 with –local -q -p options. Peaks were called with MACS2 with --macs2 callpeak -f BAMPE --nomodel --shit -100 --extsize 200 --B --SPMR -g $GENOMESIZE -q 0.01 options. Peak-associated genes were defined based on the closest genes to these genomic regions using RefSeq coordinates of genes. We used the annotatePeaks command from HOMER to calculate ATAC-seq tag densities from different experiments and to create heatmaps of tag densities. Sequencing data were visualized by preparing custom tracks for the UCSC Genome browser. De novo transcription factor motif analysis was performed with motif finder program findMotifsGenome from HOMER package on ATAC-seq peaks. Peak sequences were compared to random genomic fragments of the same size and normalized G+C content to identify motifs enriched in the targeted sequences.

For CUT&RUN analyses, data was analyzed using CUT&RUNTools pipeline (81) and resulting peak calls from SECAR were used for subsequent analysis. The peaks from IgG isotype controls were excluded in the SECAR peak calling program. A master file of H3K27me3 peaks across all the conditions and replicated was created and ncbi/BAMscale 82 was used to quantify peaks and generate scaled coverage tracks for viewing

in UCSC genome browser. The raw counts on each H3K27me3 peak were obtained using ncbi/BAMscale cov —-bed master.H3K27me3.bed –prefix Peak_count –bam *.bam command and DESeq2 was employed to obtain differential H3K27me3 peaks between F-ABC and F-FOB mice.

For IgH sequencing analyses, sequences were quality controlled with pRESTO(84). Paired reads were aligned, sequences with low quality scores were discarded, and base calls with low confidence were masked with "N"s. IgBLAST was then used to align sequences to V- and J-genes in the IMGT database(85). To group related sequences together into clones, ImmuneDB hierarchically clusters sequences with the same VH gene, same JH gene, same CDR3 length, and 85% identity at the amino acid level within the CDR3 sequence(86). Clones with consensus CDR3 sequences within 2 nucleotides of each other were further collapsed to account for incorrect gene calls. Sequencing data were submitted to SRA under project number PRJNA663307 in accordance with the MiAIRR standard(87).

For manuscripts utilizing custom algorithms or software that are central to the research but not yet described in published literature, software must be made available to editors and reviewers. We strongly encourage code deposition in a community repository (e.g. GitHub). See the Nature Research guidelines for submitting code & software for further information.

Data

Policy information about availability of data

All manuscripts must include a data availability statement. This statement should provide the following information, where applicable:

- Accession codes, unique identifiers, or web links for publicly available datasets
- A list of figures that have associated raw data
- A description of any restrictions on data availability

Sequencing data that support the findings of this study have been deposited in NCBI's Gene Expression Omnibus(GEO) with the accession code GSE175365 [https://www.ncbi.nlm.nih.gov/geo/query/acc.cgi?acc=GSE175365]. Source data are provided with this paper and are available upon request from the corresponding author.

Field-specific reporting

Please select the one below that is the best fit for your research. If you are not sure, read the appropriate sections before making your selection.

[x] Life sciences [ ] Behavioural & social sciences [ ] Ecological, evolutionary & environmental sciences

For a reference copy of the document with all sections, see nature.com/documents/nr-reporting-summary-flat.pdf

Life sciences study design

All studies must disclose on these points even when the disclosure is negative.

Sample size	The sample size was chosen based on the literature in the field. For in vivo B cell activation, previous studies by Fanzo et al. and Jordan provided the model for observing lymphocyte activation and differentiation. Their results found that approximately 60% of female mice developed lupus-like syndrome in aging Def6-deficient mice compared to none in the comparison group (p<0.005). Using those estimates, it was shown that sample sizes of five (5) achieve 95% power to detect a 60% incidence difference between the comparison groups with a significance level set at 0.05
Data exclusions	Data was not excluded from this manuscript.
Replication	Each experimental findings were reproducibles. Experimental findings were reproduced at least twice. Many of the findings were reproduced by different laboratory members at different times.
Randomization	No Randomization was used
Blinding	Blinding was used when performing histological analyses.

Reporting for specific materials, systems and methods

We require information from authors about some types of materials, experimental systems and methods used in many studies. Here, indicate whether each material, system or method listed is relevant to your study. If you are not sure if a list item applies to your research, read the appropriate section before selecting a response.

Materials & experimental systems

n/a	Involved in the study
	x Antibodies
x	Eukaryotic cell lines
x	Palaeontology and archaeology
	x Animals and other organisms
x	Human research participants
x	Clinical data
x	Dual use research of concern

Methods

n/a	Involved in the study
x	ChIP-seq
	x Flow cytometry
x	MRI-based neuroimaging

Antibodies

Antibodies used

B220-PB (RA3-6B2; 1:400; BioLegend)
B220-APC/Cy7 (RA3-6B2; 1:400; BioLegend)
B220-PE/Cy7 (RA3-6B2; 1:400; BioLegend)
BCL6-PE (K112-91; 1:100; BD)
BCL6-PE/Cy7 (K112-91; 1:100; BD)
CD4-PerCP/Cy5.5 (RM4-5; 1:400; BioLegend)
CD45-PerCP/Cy5.5 (HI30; 1:400; BioLegend)
CD11b-PE/Cy7 (M1/70; 1:400; BioLegend)
CD11b-FITC (M1/70; 1:400; BioLegend)
CD11c-APC/Cy7 (N418; 1:400; BioLegend)
CD11c-APC (N418; 1:400; BioLegend)
CD19-PB (HIB19; 1:400; BioLegend)
CD19-PE (HIB19; 1:400; BioLegend)
CD21-APC (7E9; 1:200; BioLegend)
CD23-PE (B3B4; 1:200; BioLegend)
CD23-PerCP/Cy5.5 (B3B4; 1:200; BioLegend)
CD38-PE/Cy7 (90; 1:600; BioLegend)
CD44-PE/Cy7 (IM7; 1:200; BioLegend)
CD44-A700 (IM7; 1:200; BioLegend)
CD138-APC (281-2; 1:1200; BD)
CXCR3-A488 (CXCR3-173; 1:200; BioLegend)
CXCR5-Biotin (2G8; 1:200; BD)
Fas-Biotin (Jo2; 1:200; BD)
FcRL5-FITC (509F6; 1:200; BD)
Foxp3-APC (FJK-16s; 1:100; eBioscience)
GL7-FITC (1:600; BD)
IFNg-APC (XMG1.2; 1:200; BioLegend)
IgD-FITC (11-26; 1:500; eBioscience)
IgM-PE/Cy7 (II/41; 1:1000; eBioscience)
IRF4-FITC (3E4; 1:200; eBioscience)
IRF8-PE (V3GYWCH; 1:200; eBioscience)
Ki67-PE (solA15; 1:800; eBioscience)
MerTK-APC (DS5MMER; 1:200; eBioscience)
MHC-II-PerCP/Cy5.5 (AF6-120.1; 1:600; BioLegend)
PD1-PB (J43; 1:200; eBioscience)
phospho-LYN (Y416; 1:50; Cell Signaling)
phospho-SYK (Y352; 1:50; Cell Signaling)
TACI-PE (8F10; 1:400; BioLegend)
Tbet-PE (4B10; 1:800; BioLegend)
Tbet-PE/Cy7 (4B10; 1:800; BioLegend)

Validation

All antibodies were validated by their respective companies for use in flow cytometry.

Animals and other organisms

Policy information about studies involving animals; ARRIVE guidelines recommended for reporting animal research

Laboratory animals

DEF6-deficient (Def6tr/tr) mice were generated by Lexicon Pharmaceuticals, Inc. using a gene trapping strategy. Integration of the gene-trapping construct occurred in the first intron of the Def6 gene, downstream of the exon coding for the initiation methionine. Targeted ES cells (129SvEv) were injected into C57BL/6 blastocysts to generate chimeric mice, which were then backcrossed to C57BL/6 mice for >10 generations(22). Swap70-deficient (Swap70-/-) were generated via a targeting construct designed to replace a 2.7-kb SacI/SmaI fragment containing exon 1 of the SWAP-70 gene with a phosphoglycerate kinase (PGK)-neo resistance cassette(22). Def6tr/trSwap70-/- (DKO) mice were generated by crossing Def6tr/tr mice with Swap70-/- mice that had been backcrossed onto

C57BL/6 background for >10 generations(22). C57BL/6 mice, Irf8f/f mice (#014175), and Tbx21f/f mice (#022741) were from Jackson Laboratory. B6.SB-Yaa/J were originally provided by Derry Roopenian and are available through Jackson (Yaa; #000483), Tlr7-deficient were originally provided by Eric Pamer and are available through Jackson (Tlr7-/-; #008380)(64). Male Yaa mice were crossed with female DKO mice to generate Yaa-DKO male mice(65). Irf8f/f mice, Tbx21f/f mice, and Tlr7-/- mice were crossed with DKO mice to generate Irf8f/f.DKO, Tbx21f/f.DKO mice, and Tlr7-/-.DKO mice. Generation of DKO Blimp1-YFP and Irf5f/f DKO mice were previously described(20, 66). CD23-cre mice were provided by Jayanta Chaudhuri and were previously described(67). Tbet-zsGreen-T2A-CreERT2-Rosa26-loxP-STOP-loxP-tdTomato DKO (ZTCE-DKO) mice were generated by crossing DKO mice with Tbet-zsGreen-T2A-CreERT2 provided from Jinfang Zhu(36) and with B6.Cg-Gt(ROSA)26Sortm14(CAG-tdTomato)Hze/J (#007914, Jackson).

Wild animals: This study did not involve wild animals.

Field-collected samples: This study did not involve field-collected samples.

Ethics oversight: All animal experiments were approved by the Institutional Animal Care and Use Committee of the Hospital for Special Surgery and WCMC/MSKCC and the experiments were carried out following these established guidelines.

Note that full information on the approval of the study protocol must also be provided in the manuscript.

Flow Cytometry

Plots

Confirm that:

☒ The axis labels state the marker and fluorochrome used (e.g. CD4-FITC).

☒ The axis scales are clearly visible. Include numbers along axes only for bottom left plot of group (a 'group' is an analysis of identical markers).

☒ All plots are contour plots with outliers or pseudocolor plots.

☒ A numerical value for number of cells or percentage (with statistics) is provided.

Methodology

Sample preparation: Spleen, blood, kidney, lung, or bone marrow was harvested from the indicated mice and single-cell suspensions were generated using mechanical processing and by filtering through a 40um filter. For intracellular staining, cells were fixed after surface staining at 4oC with the Transcription Factor Staining Kit (eBioscience; #00-5523-00) following the manufacturer's instructions. For intracellular cytokine staining, splenocytes were stimulated with 50g/mL PMA and 1M Ionomycin for 4hr. Cells were incubated with BrefeldinA for the final 3hr of stimulation. After stimulation, cells were fixed and permeabilized with a Transcription Factor Staining Kit (eBioscience; #00-5523-00) and stained using anti-IFN-APC (BioLegend; XMG1.2; 1:200) and recombinant mouse IL21R Fc Chimera (R&D; 1:600) followed by PE-labeled affinity-purified F(ab')2 fragment of goat anti-human Fc (Jackson ImmunoResearch). For detection of phosphorylated antigens, splenocytes were fixed in BD Fixation Buffer (#554714) for 20min at RT. Cells were then washed and permeabilized in 90% methanol for 30min at -20oC and then incubated with antibodies against phosphorylated SYK (Y352; Cell Signaling) or LYN (Y416; Cell Signaling) for 45min at room temperature.

Instrument: All data were acquired on a BD FACS Canto.

Software: All data were analyzed with FlowJo (TreeStar) software.

Cell population abundance: Purity of cell sorts were confirmed by running a small sample of sorted material immediately after sorting. Post-sort samples were between 90-98% pure.

Gating strategy: Gating strategies are shown in supplementary information and boundaries for positive and negative populations were established based on non-immunized wildtype control mice.

☒ Tick this box to confirm that a figure exemplifying the gating strategy is provided in the Supplementary Information.
